# Morbidity and mortality of Hispanic trauma patients with diabetes mellitus

**DOI:** 10.1007/s00068-018-1047-z

**Published:** 2018-11-26

**Authors:** Alanna Maria Guzman-Martinez, Omar Garcia-Rodriguez, Ediel Omar Ramos-Melendez, Lourdes Guerrios-Rivera, Pablo Rodriguez-Ortiz

**Affiliations:** 1 Department of General Surgery, University of Puerto Rico Medical Sciences Campus, San Juan, PR 00936 USA; 3 Clinical Research Office for Trauma, Puerto Rico Trauma Hospital, Río Piedras, PR 00922 USA; 2 Puerto Rico Trauma Hospital, Río Piedras, PR 00922 USA

**Keywords:** Diabetes mellitus, Hispanics, Trauma, Complications, Mortalit**y**

## Abstract

**Purpose:**

DM and trauma are leading causes of death in Hispanic patients, yet the interaction between them remains obscure. We aimed to assess the complications and in-hospital mortality rate of Hispanic diabetic trauma patients.

**Methods:**

A retrospective cohort study was carried out using data from the Puerto Rico Trauma Hospital databank. Patients were matched based on gender, age, mechanism of injury, Glasgow Coma Scale, and Injury Severity Score using propensity-score matching. From 2000 to 2014, a total of 1134 patients with DM were compared to 1134 patients who did not have DM. The outcomes measured were hospital and TICU lengths of stay, days on mechanical ventilation, complications, and in-hospital mortality rate. A logistic regression model was carried out to evaluate the relationship of DM with complications and mortality after trauma.

**Results:**

Hispanic patients with DM had longer hospital and TICU stays and required mechanical ventilation for extended periods. Complications, predominantly of an infectious nature, were more common among DM patients than they were among non-DM patients: 31.3% in the DM group vs. 11.6% in the non-DM group (OR 3.46; 95% CI 2.77–4.31). Despite an increase in the number of complications, DM was not associated with higher in-hospital mortality rates.

**Conclusions:**

DM is associated with a twofold increase in complications in Hispanic diabetic trauma patients, which may account for their longer hospital and TICU stays. This indicates that diabetic Hispanic trauma patients may need earlier and more aggressive intervention to reduce their risk of developing complications.

## Background

Diabetes mellitus (DM) is a chronic condition that affects 29.1 million people throughout the United States (US), causing a significant strain on the US economy, with estimated expenses of $176 billion in medical costs and $69 billion attributed to decreased productivity [1, 2]. Over time, DM leads to a range of complications, such as cardiovascular disease, stroke, end-stage renal disease, amputation, and visual impairment [3–5]. Trauma, also costly and common, can cause not only short- and long-term problems but also, in some cases, can lead to permanent disability; it is the leading cause of death in patients 44 years old and younger, surpassing other causes of death such as, cancer and HIV [6]. Trauma can also lead to the development of stress-induced hyperglycemia, a process shown to increase the rate of infectious complications and mortality [7–9]. Even though DM and trauma are leading causes of death, the interaction between them has not been studied in detail.

Some studies have suggested that DM increases the number of complications after trauma [10–13]. That being the case, DM patients have a greater tendency to develop infectious processes, pneumonia, and/or a myocardial infarction than do non-DM patients who have sustained similar injuries [10, 11, 14]. Looking at mortality, Liou et al. performed a retrospective cohort study on injured US patients and concluded there is a 46% excess mortality risk for insulin-dependent DM (IDDM) patients compared to such risk for patients without DM [14]. This relationship was also observed by Tebby et al. in patients with non-insulin-dependent DM (NIDDM) [15]. Similarly, injured patients with 1 or more preexisting conditions (PECs) were found to be 30% more likely to die than were those without any PECs, of which DM was one of the top 5 [14]. Conversely, other authors have not found a statistically significant association between DM and mortality after trauma [10, 11].

Another important finding is that ethnic minorities with DM residing in the US have a greater incidence of complications and increased death rates than do their non-Hispanic white counterparts [16, 17], confirming that DM affects these populations disproportionately. Trauma also has a significant impact on the US Hispanic population. In 2013, approximately 3 million US Hispanics sustained non-fatal injuries; in the same year, more than 17,000 US Hispanics died from traumatic injuries [18].

In Puerto Rico (PR), both DM and trauma were among the top 10 causes of death in 2012 [19]. Of all the states and territories of the US, PR has the highest prevalence of DM (12.7%) [20, 21]; compared to all the countries in the world, PR has the second greatest number of DM-related deaths [19]. In PR, the demographics of trauma patients are changing, as adults aged 65 and older are now the fastest growing segment of the population and younger individuals are migrating to the US mainland. The high prevalence and mortality disparity of these 2 diseases, along with the lack of data and the inconsistency of the available scientific evidence, combine to illustrate the need for further investigation of this public health threat. Therefore, we aimed to evaluate the relationship between DM and trauma outcome, particularly the effects of that relationship on complications and in-hospital mortality rate.

## Methods

A retrospective cohort study was carried out at the Puerto Rico Trauma Hospital (PRTH) to fulfill the aims of this project. The PRTH is the only acute-care institution serving the citizens of PR and the Caribbean and its trauma bay receives over 1500 patients per year. The electronic records of 22,123 patients treated at the PRTH from January 2000 to December 2014 were retrieved from the Trauma Registry of the hospital, a division of the US National Trauma Registry System (a databank monitored by the American College of Surgeons). The inclusion criteria consisted of having a known diagnosis of DM, as determined using the International Classification of Diseases, Ninth Revision (ICD-9), and being of Hispanic origin, as reported by patients on their admission records. A total of 1134 exposed (diabetic) subjects were found to meet the criteria.

Following the selection of the DM patients, subjects were paired with 1134 non-DM (unexposed) patients, utilizing a propensity index calculated with the use of a logistic regression model. Propensity score matching is a method that matches patients based on similar baseline characteristics. A score is given to each individual and that score is used to pair up (in this case) a diabetic trauma patient with a non-diabetic trauma patient. This procedure is performed to eliminate the possibility of selection bias [22]. The categorical variables matched were sex, age, mechanism of trauma, Glasgow Coma Scale (GCS) score, and Injury Severity Score (ISS); patients whose records lacked these data were excluded.

To estimate the statistical power (88%) of our study, we assumed an 11% trauma mortality rate for unexposed subjects [23] and an odds ratio (OR) of 1.46 [14], with a sample size of 1134 subjects in each cohort and a confidence level of 95%.

The demographic data collected consisted of age (number of years old), and sex (male/female). The clinical information gathered included the following variables: admission vital signs, mechanism of injury [fall, motor vehicle collision (MVC), gunshot wound, pedestrian vs. motor vehicle, or other], area(s) of injury (head/neck, chest, abdomen, and/or extremity), days on mechanical ventilator (MV), hospital length of stay (LOS), number of days in the trauma intensive care unit (TICU), and complications developed during the stay (infectious and noninfectious). We were unable to include the glycemic and HbgA1C levels of DM patients, as this clinical information was not consistently available in the records of patients selected for our study. The primary outcome measures were complications and in-hospital mortality.

Descriptive statistics, including the median and interquartile range, as well as the absolute (*n*) and relative (%) frequencies, were measured to assess the continuous and categorical variables, respectively. To compare the DM group to the non-DM group, categorical variables were further analyzed with Pearson’s Chi square tests and continuous variables with Mann–Whitney tests. To determine whether Hispanic trauma patients with DM are at greater risk for developing complications and for mortality than are those without DM, a binary logistic regression analysis was completed.

Statistical analysis was carried out with Stata Statistical Software: Release 14 for Windows. A *p* value lower than 0.05 was an indication of statistical significance. Approval of the study protocol was obtained from the Institutional Review Board of the Medical Sciences Campus of the University of Puerto Rico, prior to commencing data collection.

## Results

Hispanic DM patients were predominantly males (75.3%); the majority age group was that comprising individuals ranging in age from 41 to 64 years old (45.5%). On admission, a GCS score of less than 9 was observed in 6.4% of the sample members and an ISS greater than 24 was observed in 18.7% of patients. The most common mechanism of injury was MVC (37.9%). Regarding area of injury, DM patients were more likely than their non-DM counterparts to suffer abdominal trauma (26.5% vs. 22.1%; *p* = 0.016) and injuries to the chest (50.6% vs. 39.8%; *p* < 0.001). On the other hand, non-DM patients had a greater incidence of trauma to the extremities, compared to such incidence for DM patients (51.9% in the DM group vs. 57.5% in the non-DM group; *p* = 0.008) (Table 1).

**Table 1:** Socio-demographic variables of DM and non-DM trauma patients

Characteristic	DM (*n* = 1134)	Non-DM (*n* = 1134)	*p* value
Sex			
Male	854 (75.3)	854 (75.3)	Matched
Female	280 (24.7)	280 (24.7)	
Age (years)			
< 18	17 (1.5)	17 (1.5)	Matched
18–40	166 (14.6)	166 (14.6)	
41–64	516 (45.5)	516 (45.5)	
> 64	435 (38.4)	435 (38.4)	
Mechanism of injury			
Fall	134 (11.8)	134 (11.8)	Matched
MVC	429 (37.8)	430 (37.9)	
Gunshot wound	70 (6.2)	70 (6.2)	
Stab wound	47 (4.1)	46 (4.0)	
Pedestrian	160 (14.1)	160 (14.1)	
Other	294 (25.9)	294 (25.9)	
GCS Score			
13–15	1024 (90.3)	1024 (90.3)	Matched
9–12	37 (3.26)	37 (3.26)	
≤ 8	73 (6.44)	73 (6.44)	
ISS Score			
1–9	485 (42.8)	486 (42.9)	Matched
10–15	161 (14.2)	159 (14.0)	
16–24	276 (24.3)	278 (24.5)	
≥ 25	212 (18.7)	211 (18.6)	
Area of injury			
Head/Neck < 2	845 (74.5)	808 (71.3)	0.081
Head/Neck ≥ 2	289 (25.5)	326 (28.7)	
Chest < 2	560 (49.4)	683 (60.2)	< 0.001
Chest ≥ 2	574 (50.6)	451 (39.8)	
Abdomen < 2	834 (73.5)	883 (77.9)	0.016
Abdomen ≥ 2	300 (26.5)	251 (22.1)	
Extremities < 2	545 (48.1)	482 (42.5)	0.008
Extremities ≥ 2	589 (51.9)	652 (57.5)	
SBP			
≥ 90	1067 (95.1)	1025 (91.0)	< 0.001
< 90	55 (4.9)	102 (9.1)	
Heart rate			
Normal	703 (62.3)	791 (70.2)	< 0.001
Bradycardia	43 (3.8)	45 (4.0)	
Tachycardia	382 (33.9)	291 (25.8)	
Respiratory rate			
Normal	705 (64.8)	652 (58.5)	0.005
Hypoventilation	6 (0.6)	13 (1.2)	
Hyperventilation	377 (34.7)	449 (40.3)	

The results are expressed as *n* (%)

*MVC* motor vehicle collision, *GCS* Glasgow coma scale, *ISS* injury severity score, *SBP* systolic blood pressure

On admission, a lower proportion of DM patients had SBP of less than 90 (4.9% vs. 9.1%; *p* < 0.001) and hyperventilation (34.7% vs. 40.3%; *p* = 0.005), compared to their non-DM counterparts. Conversely, a higher number of DM patients than non-DM patients had tachycardia (33.9% vs. 25.8%; *p* < 0.001) (Table 1). The median number of days in the hospital (12 days for the DM group vs. 6 days for the non-DM group; *p* < 0.001) and in the TICU (17 days for the DM group vs. 9 days for the non-DM group; *p* < 0.001), as well as days on MV (15 days for the DM group vs. 6 days for the non-DM group; *p* < 0.001), were all significantly greater for DM patients (Table 2).

**Table 2:** Survival, length of stay and days on mechanical ventilator between DM and non-DM patients post-trauma

Characteristic	DM (*n* = 1134)	Non-DM (*n* = 1134)	*p* value
Probability of survival	0.97 (0.05)	0.98 (0.05)	< 0.001
Hospital LOS	12 (19)	6 (11)	< 0.001
TICU LOS	17 (21)	9 (8)	< 0.001
MV days	15 (22)	6 (12)	< 0.001

The results are expressed as median (interquartile range)

*LOS* length of stay, *TICU* trauma intensive care unit, *MV* mechanical ventilator

Our diabetic and non-diabetic trauma-patient population had a series of comorbidities, the top 3 being hypertension (60% vs. 7.2%; *p* < 0.001), heart disease (8.8% vs. 1.6%; *p* < 0.001), and respiratory disorder (2.6% vs. 0%; *p* < 0.001) (Table 3).

**Table 3:** Comorbidities of DM and non-DM trauma patients

Comorbidity	DM (*n* = 1134)	Non-DM (*n* = 1134)	*p* value	OR (95% CI)^a^
Hypertension	680 (60.0)	82 (7.2)	< 0.001	19.22 (14.90–24.78)
Heart disease	100 (8.8)	18 (1.6)	< 0.001	6.00 (3.60–9.97)
Respiratory disease	30 (2.7)	0 (0.0)	< 0.001	–
Obesity	28 (2.5)	3 (0.3)	< 0.001	9.54 (2.89–31.48)
Myocardial infarction	21 (1.9)	7 (0.6)	0.008	3.04 (1.29–7.17)

The results are expressed as *n* (%) and OR (95% CI)

*OR* Odds ratio, *CI* confidence interval

^a^Non-DM patients were used as reference category

Regarding complications, our results indicate that diabetic trauma patients were 6 times more likely to develop wound infections than were non-diabetic trauma patients (OR 6.20; 95% CI 2.22–17.35). Urinary tract infections were 2 times more common in diabetics than non-diabetics (OR 2.05; 95% CI 1.29–3.23), and septicemia and pneumonia were nearly 2 times more likely to occur in DM patients than non-DM patients (OR 1.70; 95% CI 1.02–2.82 and OR 1.60; 95% CI 1.02–2.51) (Table 4).

**Table 4:** Complications associated with DM and non-DM trauma patients

Complication	DM (*n* = 1134)	Non-DM (*n* = 1134)	*p* value	OR (95% CI)^a^
Infectious				
Urinary tract infections	107 (9.4)	35 (3.1)	< 0.001	2.05 (1.29–3.23)
Septicemia	61 (5.4)	32 (2.8)	0.002	1.70 (1.02–2.82)
Bacteremia	49 (4.3)	20 (1.8)	< 0.001	1.17 (0.62–2.24)
Wound infection	30 (2.7)	5 (0.4)	< 0.001	6.21 (2.22–17.35)
Pulmonary				
Pneumonia	108 (9.5)	40 (3.5)	< 0.001	1.60 (1.02–2.51)
ARDS	57 (5.0)	58 (5.1)	0.924	1.14 (0.73–1.78)
Hemothorax	39 (3.4)	25 (2.2)	0.076	1.16 (0.62–2.17)
Pleural effusion	36 (3.2)	4 (0.4)	< 0.001	7.44 (2.45–22.61)
Pulmonary embolism	11 (1.0)	33 (2.9)	0.001	0.28 (0.12–0.64)
Cardiovascular				
Cardiac arrest	40 (3.5)	7 (0.6)	< 0.001	4.50 (1.86–10.87)
Arrhythmia	24 (2.1)	11 (1.0)	0.027	0.98 (0.41–2.31)
Myocardial infarction	5 (0.4)	6 (0.5)	0.762	0.78 (0.19–3.24)
Others				
Renal failure	64 (5.6)	18 (1.6)	< 0.001	2.11 (1.13–3.96)
Decubitus ulcer	25 (2.2)	2 (0.2)	< 0.001	6.90 (1.49–31.96)
Compartment syndrome	10 (0.9)	14 (1.2)	0.412	0.12 (0.05–0.32)
Coagulopathy	2 (0.2)	23 (2.0)	< 0.001	0.01(0.003–0.065)

The results are expressed as *n* (%) and OR (95% CI)

*OR* Odds ratio, *CI* confidence interval, *ARDS* acute respiratory distress syndrome

^a^Non-DM patients were used as reference category

Despite the increased rate of complications, there was no significant difference in mortality rates between DM and non-DM patients (OR 1.08; 95% CI 0.87–1.36).

## Discussion

Trauma and DM are leading causes of death in the Hispanic population. Despite this fact, the few studies that have evaluated the relationship between them have consisted mostly of Caucasian patients, with the Hispanic patients studied being aggregated with other minorities. Therefore, our goal was to evaluate the complications generally suffered by Hispanic DM trauma patients and the in-hospital mortality rate of this group. Our findings confirm the result of previous studies that also found that DM patients had a greater tendency than did their non-DM counterparts to develop infectious complications after trauma [10]. However, the number of infectious complications suffered by Hispanic DM trauma patients who took part in our study was significantly greater than what has been reported in previous studies, in which Hispanic DM patients were in the minority. In our study, complications, predominantly of an infectious nature, were more common among DM patients than they were among non-DM patients: 31.3% in the DM group vs. 11.6% in the non-DM group (OR 3.46; 95% CI 2.77–4.31).

Ahmad et al. performed a retrospective analysis of 12,489 patients (65% of whom were male) who were hospitalized due to injury from 1984 to 2002. They evaluated cases from the Pennsylvania Trauma Systems Foundation database and matched the DM patients with an equal number of non-DM patients. Ahmad and his team reported a compelling difference in the rate of complications between the DM and the non-DM groups: Twenty-three percent of their DM patients developed infectious complications vs. 14% of their non-DM patients, who did not. Infectious complications such as wound infections and pneumonia were less than 2 times more common in diabetics compared to non-diabetics [10]. A stark difference is seen in our results, in which Hispanic DM patients were 6 times more likely to develop wound infections than were non-DM patients. Similarly, Kao et al. evaluated 343,250 patients from the US National Trauma Data Bank who were injured from 1994 to 2003 and of which number only 2.7% were diabetic. In their analysis, they noted that insulin-dependent DM patients had a 58% excess risk of developing infectious complications compared to the risk of same sustained by non-DM patients [11].

In respect to ICU stay and need for mechanical ventilator, Ahmad et al. showed that patients with DM had extended ICU stays compared to those of the non-DM group (7.6 days in the DM group vs. 5.0 in the non-DM group). The patients in their DM group also received ventilator support for a greater period of time than did their non-DM group (10.7 days in the DM group vs. 6.7 days in the non-DM group) [10]. Even though our results are similar to those of this previous study, the difference in TICU LOS and days on MV was 2 times greater in our DM patients when compared to the results presented by Ahmad et al. [10] (Our median TICU LOS was 17 days in the DM group vs. 9 days in the non-DM group and the need for MV was 15 days in the DM group vs. 6 days in the non-DM group). This could indicate that our population of Hispanic DM patients have a significantly greater risk of developing complications than Caucasian DM patients, which is a novel finding in the trauma setting.

The presence of PECs may be responsible for the relatively higher morbidity observed in DM patients. In fact, a study conducted by Morris et al. demonstrated that a group of patients in the state of California who had been injured in 1983 and who had 1 or more PECs were 30% more likely to die than were patients without any PECs, with DM being one of the top 5 PECs in their analysis [14]. This suggests that DM affects not only a patient’s baseline health status but also his or her recovery process. Even though our study observed comorbidities similar to those noted by Morris et al. [14], there were differences in terms of mortality.

The excess morbidity observed in DM patients following trauma may be attributed to changes in the immune system that are a consequence of the inciting injury, which may be reflected by a given patient’s elevated glycemic level [10]. Trauma across all ages is associated with a metabolic response known as stress-induced hyperglycemia, in which the body releases catecholamine and stress hormones, causing an elevation in blood sugar [24]. Hyperglycemia leads to impairment of chemotaxis and adherence, oxidative bursts, and the death of polymorphonuclear cells, all of which can lead to the inability to combat infections [25–28]. This process has been linked to increased TICU and hospital LOS [7, 8, 29], rates of infectious complications, and mortality [7–9].

Our study determined that diabetic Hispanic trauma patients had a twofold increase in the rate of complications, compared to the rate observed in Caucasian diabetic trauma patients. Strategies to identify these patients upon arrival at the trauma bay may decrease in-hospital complications and LOS. For example, the HbA1C levels of all the diabetic patients could be measured automatically as part of that patient’s initial work-up; in this way, health care personnel could determine whether the patient’s DM was controlled or uncontrolled. Strict measurement of glucose levels in the early stages post-injury could also be implemented. This could lead to an earlier and more aggressive management of that individual’s DM and, in turn, fewer complications. A prospective randomized trial performed by Van den Berghe demonstrated a relationship between intense insulin therapy (keeping glucose levels < 200 mg/dL) and a reduction in the morbidity and mortality of diabetic surgical ICU patients [30]. This has yet to be proven in the diabetic trauma patient; therefore, our study would serve as the initial step in establishing an insulin regimen for diabetic trauma patients, especially in the Hispanic patient population, which is disproportionately affected by diabetes.

As to mortality, our results indicate that there is no statistically significant difference in mortality when DM patients are compared to non-DM patients. The scientific literature is inconsistent regarding the role of DM in trauma mortality. For instance, Tebby et al. found an excess mortality risk of 64% for diabetics, after adjusting for age, sex, ISS, GCS score, and PECs [12]. Meanwhile, Liou et al. determined that IDDM patients have a 46% excess mortality risk compared to patients without DM [15]. However, this relationship was not observed in the NIDDM group. This fact is of particular importance because when these two categories are collapsed (IDDM and NIDDM), the effect that DM has on trauma mortality is diluted.

Other researchers, however, have reported findings similar to those documented by us. In Ahmad and colleagues’ study, the mortality analysis was performed matching DM patients with non-DM patients by sex, age, and ISS to allow comparisons of similar groups [10]. With a total of 24,978 participants for this sub-analysis, the authors did not find statistically significant differences for mortality between the compared groups. Furthermore, when Kao et al. evaluated the hypothesis that DM results in a higher mortality in trauma patients, they did not find a statistically significant association, after adjusting for age, ISS, and mechanism of injury [11]. The authors also entered the variables IDDM and NIDDM into the mortality prediction model as separate terms, and neither was statistically significant.

These four studies confirm the heterogeneity that exists in the evidence produced on diabetes and trauma mortality. All these investigations included sophisticated analyses that, in some way, considered potential confounding variables. In our case, we decided to control the potential confounding variables in our design phase using a propensity score matching technique to match patients based on strong mortality predictors such as sex, age, mechanism of injury, GCS score, and ISS. The study by Ahmad et al. [10], who also used the matching technique, found no difference in mortality. Therefore, the method selected to control confounding variables, as well as the populations in which the studies were conducted, might explain the differences found in the studies on this topic.

Our study has several limitations, including its retrospective nature, which may have led to misclassification bias. Because we used ICD-9 codes to identify the DM patients, there exists the possibility that we might have missed patients who did not have an established diagnosis of DM on admission. As a result, undiagnosed patients may have been included in our non-DM group.

An important limitation within our study was the inability to include the glycemic and HbgA1C levels of DM patients, as this clinical information was not consistently available in the records of all the patients selected for our study. Despite differences in the pathophysiology of DM types 1 and 2, DM status also could not be further subdivided, and therefore, differences in the outcomes of such patients could not be analyzed. Another limitation of our database was that it lacked certain variables, such as admission glucose levels, body mass index, medication compliance, and time since diagnosis of DM.

These limitations are balanced by the key strengths of our study, the primary being that it focused on Hispanic trauma patients, a population that has not been previously studied on its own. Considering the lack of knowledge in this area, there was a need to determine if there are any differences in terms of morbidity and mortality in this patient population, as it is the largest minority group in the US. Our study also analyzed data collected over an extended period of time, which adds consistency to our findings.

## Conclusion

Our study determined that, although Hispanic diabetic trauma patients do not have a significant difference in mortality, they have a twofold increased rate of complications, compared to the rate observed in Caucasian diabetic trauma patients. Strategies to identify such patients upon arrival at the trauma bay are necessary to decrease in-hospital complications among these patients. For example, the HbA1C levels of all DM patients could be measured to determine if a patient is a controlled or an uncontrolled DM patient. This could lead to an earlier and more aggressive management of DM and, as a result, fewer complications and lower TICU/hospital LOS. The conflicting findings of mortality in trauma DM patients may be due to the effect of confounding variables, which we believe we minimized in our study by matching patients using strong mortality predictors. Distinct patient characteristics and baseline health status may also account for the difference in mortality between Caucasian and Hispanic diabetic trauma patients.
